# Increased metabolic variability in Korean patients with new onset bipolar disorder: a nationwide cohort study

**DOI:** 10.3389/fpsyt.2023.1256458

**Published:** 2024-01-08

**Authors:** Ji Hyun Baek, Kyungdo Han, Hyewon Kim, Kyojin Yang, Hong Jin Jeon

**Affiliations:** ^1^Department of Psychiatry, Depression Center, Samsung Medical Center, Sungkyunkwan University School of Medicine, Seoul, Republic of Korea; ^2^Dauten Family Center for Bipolar Treatment Innovation, Massachusetts General Hospital, Boston, MA, United States; ^3^Department of Statistics and Actuarial Science, Soongsil University, Seoul, Republic of Korea; ^4^Department of Health Sciences & Technology, Department of Medical Device Management & Research, and Department of Clinical Research Design & Evaluation, Samsung Advanced Institute for Health Sciences & Technology (SAIHST), Sungkyunkwan University, Seoul, Republic of Korea

**Keywords:** bipolar disorder, metabolic variability, metabolic syndrome, old age onset bipolar disorder, modifiable risk factor

## Abstract

**Introduction:**

The aim of this study was to determine associations between changes of metabolic parameters and the development of BD using nationally representative data.

**Methods:**

We used health examination data provided by the South Korean National Health Insurance System (NHIS) (*n* = 8,326,953). The variability of each metabolic parameter including weight circumference, blood pressure, fasting blood glucose, high-density lipoprotein cholesterol, and triglyceride levels was caculated using variability independent of mean (VIM) indices. The presence of metabolic syndrome was associated with new onset BD. Each metabolic parameter with high variability was associated with a higher risk of new onset BD compared to those with low variability after adjusting for age, sex, smoking, alcohol drinking, regular exercise, income status, baseline diabetes, hypertension, and dyslipidemia.

**Results:**

As the number of highly variable metabolic parameters increased, the risk for new onset depression also increased even after covariates adjustment. The associations between new onset BD and metabolic variability were greater in populations with age > 50 years. In addition, these associations remained significant after adjusting for the presence of depression prior to diagnoses of BD.

**Discussion:**

Our results suggest possibility of metabolic variability as an independent environmental risk factor for BD even after adjusting for the presence of metabolic syndrome.

## 1 Introduction

Bipolar disorder (BD) is a chronic mental disorder with a high disease burden. BD is a highly heritable disease, although about 15–40% of risk is derived from environmental factors (1). In general, BD develops from complex interplays between genetic and environmental factors. Obstetric complication, infections, and adverse childhood experiences have been suggested as potential environmental risk factors (2). However, little is known if there are modifiable environmental factors for the development of BD.

Metabolic parameters could be one of the potential environmental factors associated with the development of BD. The association between BD and metabolic syndrome is complex. Metabolic syndrome is quite common in BD. Treatment agents for BD could increase the risk of metabolic syndrome. However, metabolic syndrome is common even in drug naïve patients with BD. Based on these findings, shared genetic and pathophysiological mechanisms have been suggested (3, 4). Metabolic syndrome is associated with worse clinical features including chronic course of illness, worse global functioning, and rapid cycling in BD (5). Metabolic syndrome also affects cognitive functions. In particular, metabolic syndrome can cause structural and functional changes in the brain (6), which could be linked to the pathophysiology of BD.

Metabolic parameters could be more closely associated with late-onset BD defined as BD that develops >50 years old (7). It has been reported that late-onset BD has weaker associations with family history of BD than early onset BD, but has greater associations with neurological conditions and other somatic problems (8).

We have previously reported that metabolic variability defined as dynamic changes of metabolic parameters is associated with new onset depression and the presence of metabolic syndrome *per se* using Korean nationwide data (9). Several studies has reported associations of metabolic syndrome with new onset BD (10–13), but they did not use nationally representative data. In addition, no prior study examined the association between metabolic variability and new onset BD. Therefore, the objective of this study was to explore associations of metabolic syndrome and metabolic variability with the development of BD using nation-wide data in Korea.

## 2 Methods

### 2.1 Data source and study population

Details of data source and the study population were described in our previous study (9). In brief, we used nationally representative data from the Korean national health insurance service (NHIS) (14). The NHIS is the single insurer managed by the Korean government and covers all Koreans. All people who enrolled in the NHIS are recommended to receive standardized medical examinations every 2 years. Data on sociodemographic characteristics, a medical treatment history, a health examination results and death information as de-identified and anonymous format are available in the NHIS database.

**Figure 1:**
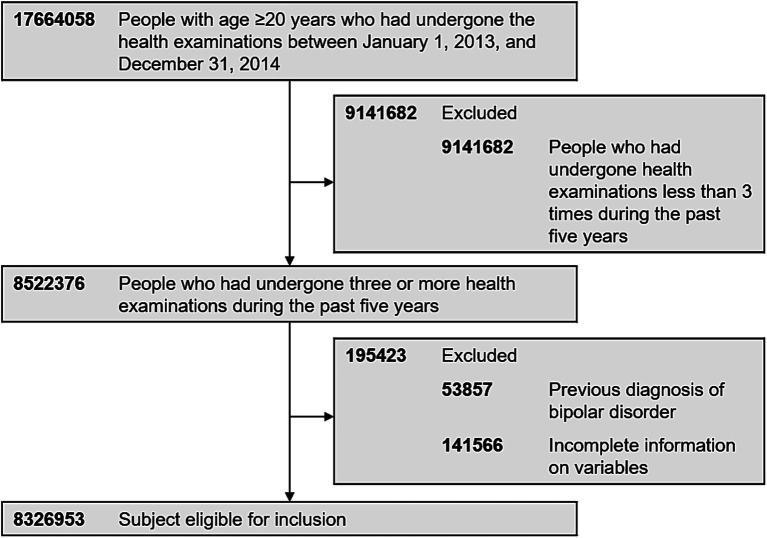
Flow chart of study participants.

Inclusion criteria for this study was those who had undergone the health examinations provided by the South Korean NHIS between January 1, 2013, and December 31, 2014 (index year). Of 17,664,058 people with age ≥ 20 years with health examination data in the index year, 8,522,376 underwent three or more health examinations during the past 5 years (including 2013 and 2014). Exclusion criteria was those who had a history of BD (International Classification of Disease, 10th Revision [ICD-10] codes: F30 and F31) before the index year (*n* = 53,857). Individuals with missing data for one or more variables were also excluded in the analyses. A total of 8,326,953 people were finally included in the analyses (Figure 1). This study was approved by the Institutional Review Board (IRB) of Samsung Medical Center (IRB No. 2020–10-005). We did not have an informed consent because all data was de-identified before they became publicly available.

### 2.2 Measurements

The detailed general medical examination process and definitions of each medical condition were described elsewhere (15). Each hospital where the health examinations were took place had certification by the NHIS for the quality control. In brief, it includes medical history taking, vital sign check-up and blood sampling. A trained clinician measured blood pressure after at least 5 min’ rest with the arm at an appropriate position. Blood samples were collected after an overnight fast. Measurements included in the blood analysis were total cholesterol, triglyceride, high-density lipoprotein (HDL) and low-density lipoprotein (LDL) cholesterol, hemoglobin, serum creatinine, aspartate aminotransferase (AST), alanine transaminase (ALT), gamma glutamyl transferase (GTP) (14).

The diagnosis of diabetes mellitus (DM) was defined according to the presence of ≥1 claim per year under ICD-10 codes (I11–14) and ≥ 1 claim per year for the prescription of antidiabetic medication or fasting glucose levels ≥126 mg/dL. The diagnosis of hypertension was defined according to the presence of ≥1 claim per year under ICD-10 codes (I10–13 or I15) and ≥ 1 claim per year for the prescription of antihypertensive agents or systolic/diastolic BP of ≥140 mmHg/90 mmHg.

We defined the diagnosis of dyslipidemia as the presence of ≥1 claim per year under ICD-10 code E78 and ≥ 1 claim per year for the prescription of a lipid-lowering agent or a total cholesterol (TC) level ≥ 240 mg/dL. Obesity was defined as a body mass index (BMI) ≥ 25 kg/m^2^ (16) according to the Korean classification. We used survey data in evaluating smoking and alcohol consumption history. Heavy alcohol consumption was defined as ≥30 g/day. Regular exercise was defined as performing >30 min of moderate physical activity ≥5 times per week or > 20 min of strenuous physical activity ≥3 times per week. Low household income level was defined as the income level below 20%.

We used the National Cholesterol Education Program Adult Treatment Panel guideline (NCEP-ATP III) definition (17) to define metabolic syndrome.

### 2.3 Metabolic variability

We used the same definitions for metabolic variability with those used in our previous study (9). In brief, metabolic variability was defined as intra-individual variability of metabolic variables recorded in health examinations. We used variability independent of the mean as in previous studies (15). Variability independent of the mean was calculated by dividing the standard deviation (SD) by the mean to the power *x*. Power *x* was modeled as SD = *k* × mean*^x^*. It was derived from fitting curves by nonlinear regression analysis. For the analyses, we used the PROC NLIN procedure of the SAS package (18).

Highly variable metabolic parameters were defined as the highest quartile (Q4) of the variability. Low variability was defined as the lower three quartiles (Q1– Q3) of variability. Participants were classified further based on the number of high-variability metabolic parameters (waist circumference, blood pressure, triglyceride, HDL, and fasting blood sugar levels) using a score ranging from 0 to 5, meaning a score of 0 indicated no high-variability parameter, 1 to 5 indicated the number of high-variability parameters out of a total of five parameters.

### 2.4 Study outcomes and follow-up

The primary outcome measure was the new diagnosis of BD during the follow-up period. New onset BD was defined by a primary diagnosis of BD (F30 and F31) based on the International Statistical Classification of Disease and Related Health Problems 10th revision (ICD-10). The study population was followed from baseline to the date of new onset BD, death, or until December 31, 2018, whichever came first.

### 2.5 Previous history of depression

Because we used the diagnostic code based on the ICD-10 when defining new onset BD, there could be patients previously diagnosed with depression (F32 and F33) who later had a diagnosis of BD (F30 and F31). Considering this as a diagnostic conversion from unipolar depression to BD within mood spectrum disorder, we conducted subgroup analysis by dividing study participants into two groups based on history of depression prior to examination. A previous history of depression was defined as a primary diagnosis of depressive disorder (F32 and F33).

### 2.6 Statistical analysis

Continuous variables were presented as mean ± standard deviation (SD), while categorical variables were presented as n (%). Participants were classified into six groups based on the number of high-variability metabolic variables. Characteristics of these six groups were compared. The incidence rate of the primary outcome was calculated by dividing the number of incident cases by the total follow-up duration (person-years). The cumulative incidence of new onset BD according to the number of parameters with high variability was presented using unadjusted Kaplan–Meier curves. We conducted the log-rank test to analyze differences between groups. Hazard ratio (HR) and 95% confidence interval (CI) for new onset BD were analyzed using the Cox proportional-hazards model. A multivariable-adjusted proportional-hazards model was applied. Model 1 was not adjusted. Model 2 was adjusted for age and sex. Model 3 was adjusted for age, sex, smoking, alcohol consumption, regular exercise, and income status. Model 4 was adjusted for age, sex, smoking, alcohol consumption, regular exercise, low-income status, baseline diabetes, hypertension, and dyslipidemia. We additionally explored whether the number of metabolic parameters with high variability showed different effects on the development of BD depending on gender and age group. All statistical analyses were performed using SAS version 9.4 (SAS Institute Inc). A *p*-value of less than 0.05 indicated significance.

## 3 Results

### 3.1 Baseline characteristics

Table 1 shows characteristics of participants. Participants with greater numbers of highly variable metabolic parameters (metabolic parameters in the 4th quartile of variability) were older and more likely to be females. Those with greater numbers of highly variable metabolic parameters had diagnoses of hypertension, DM, and dyslipidemia more frequently than those with fewer highly variable metabolic parameters. They also smoked less and drink less. They performed less regular exercise. They had low household income more frequently. Those with greater numbers of highly variable metabolic parameters more frequently had metabolic syndrome (≥ 3 metabolic parameters met the criteria for metabolic syndrome (MS)), while those with fewer highly variable metabolic parameters had MS less frequently (*p* < 0.001 for all variables displayed in Table 1).

**Table 1:** Baseline characteristics of subjects according to the number of highly variable metabolic parameters.

Variable	Number of highly variable metabolic parameters					
	0	1	2	3	4	5
N	2,187,047	3,084,330	2,057,212	800,920	179,566	17,878
Age						
< 40 yrs	622,410 (28.46)	860,358 (27.89)	554,883 (26.97)	208,500 (26.03)	45,713 (25.46)	4,350 (24.33)
40–64 yrs	1,328,104 (60.73)	1,813,257 (58.79)	1,172,609 (57)	443,025 (55.31)	95,787 (53.34)	9,275 (51.88)
≥ 65 yrs	236,533 (10.82)	410,715 (13.32)	329,720 (16.03)	149,395 (18.65)	38,066 (21.2)	4,253 (23.79)
Sex, male	1,351,145 (61.78)	1,808,960 (58.65)	1,178,149 (57.27)	451,025 (56.31)	99,002 (55.13)	9,727 (54.41)
Body mass index (BMI), kg/m^2^	23.7 ± 3.1	23.84 ± 3.22	24.02 ± 3.32	24.18 ± 3.41	24.31 ± 3.54	24.44 ± 3.66
Waist circumference, cm	80.17 ± 8.79	80.64 ± 9.07	81.29 ± 9.27	81.92 ± 9.47	82.46 ± 9.79	82.95 ± 9.98
Fasting blood glucose, mg/dl	96.91 ± 17.73	97.78 ± 21.06	99.12 ± 24.79	100.75 ± 28.84	102.49 ± 32.84	104.57 ± 36.52
Systolic blood pressure, mmHg	121.74 ± 12.69	121.81 ± 13.9	122.18 ± 14.95	122.54 ± 15.99	122.7 ± 17.1	123 ± 18.6
Diastolic blood pressure, mmHg	76.11 ± 9.15	76.02 ± 9.53	76.12 ± 9.9	76.18 ± 10.28	76.12 ± 10.75	76.14 ± 11.42
Total cholesterol, mg/dL	197.21 ± 34.86	195.64 ± 35.8	194.19 ± 37.01	192.75 ± 38.35	191.18 ± 39.78	189.89 ± 41.97
HDL cholesterol, mg/dL	56.49 ± 13.86	55.43 ± 15.73	54.05 ± 16.65	52.61 ± 17.6	51.28 ± 17.02	50.15 ± 16.56
LDL cholesterol, mg/dL	116.51 ± 32.87	114.91 ± 33.84	113.45 ± 34.83	112.05 ± 36.08	110.56 ± 36.91	109.3 ± 38.48
Triglyceride, mg/dL	4.67 ± 0.52	4.7 ± 0.56	4.74 ± 0.59	4.79 ± 0.62	4.82 ± 0.65	4.84 ± 0.68
Current smoker, yes	533,453 (24.39)	763,598 (24.76)	519,742 (25.26)	204,667 (25.55)	45,628 (25.41)	4,497 (25.15)
Heavy alcohol drinker, yes	159,050 (7.27)	220,847 (7.16)	148,204 (7.2)	58,006 (7.24)	13,064 (7.28)	1,277 (7.14)
Regular exercise	488,689 (22.3)	673,615 (21.8)	439,830 (21.4)	167,218 (20.9)	36,187 (20.2)	3,582 (20.0)
Household income lower 20%	334,344 (15.3)	530,770 (17.2)	384,158 (18.7)	159,184 (19.9)	37,780 (21.0)	4,014 (22.5)
Metabolic syndrome, yes	476,951 (21.8)	813,293 (26.4)	652,044 (31.7)	299,314 (37.4)	76,934 (42.8)	8,596 (48.1)
Number of metabolic parameters that met the definition of metabolic syndrome						
0	685,759 (31.4)	828,256 (26.9)	453,084 (22.0)	141,424 (17.7)	25,562 (14.2)	2,024 (11.3)
1	595,024 (27.2)	808,552 (26.2)	508,389 (24.7)	183,290 (22.9)	37,536 (20.9)	3,495 (19.6)
2	429,313 (19.6)	634,229 (20.6)	443,695 (21.6)	176,892 (22.1)	39,534 (22.0)	3,763 (21.1)
3	277,327 (12.7)	449,802 (14.6)	342,747 (16.7)	149,302 (18.6)	36,438 (20.3)	3,746 (21.0)
4	151,991 (7.0)	270,034 (8.8)	225,257 (11.0)	107,349 (13.4)	28,237 (15.7)	3,297 (18.4)
5	47,633 (2.2)	93,457 (3.0)	84,040 (4.1)	42,663 (5.3)	12,259 (6.8)	1,553 (8.7)

Data are expressed as mean ± SD (standard deviation) or n (%). *P*-values for trend < 0.0001 for all variables because of the large size of the study population.

HDL, high-density lipoprotein; LDL, low-density lipoprotein; BMI, body mass index; yrs, years.

### 3.2 Risk of BD by metabolic syndrome and its components

Table 2 displays hazard ratio of new onset BD in the presence of metabolic syndrome. There were a total of 11,027 cases of new onset BD during a mean follow-up of 4.22 years (standard deviation (SD): 0.5 years) in the entire cohort. The presence of metabolic syndrome was associated with the development of BD even after adjusting for aforementioned covariates (HR: 1.16, 95% CI: 1.11–1.21). The presence of the condition that met the criteria for each criterion of metabolic syndrome was also independently associated with the development of depression (HR: 1.05–1.26).

**Table 2:** Hazard ratios and 95% confidence intervals of new onset bipolar disorder in the presence of metabolic syndrome.

	*N*	New onset bipolar disorder (N)	Follow-up duration (person-year)	Incidence rate (per 1,000)	Model 1	Model 2	Model 3	Model 4
Presence of Metabolic syndrome								
No	5,999,821	7,061	25,270,363.13	0.27942	1 (Ref.)	1 (Ref.)	1 (Ref.)	1 (Ref.)
Yes	2,327,132	3,966	9,838,903.27	0.40309	1.434 (1.38,1.491)	1.088 (1.044,1.133)	1.081 (1.038,1.126)	1.16 (1.109,1.212)
Weight circumference								
No	6,610,635	8,374	27,874,323.73	0.30042	1 (Ref.)	1 (Ref.)	1 (Ref.)	1 (Ref.)
Yes	1,716,318	2,653	7,234,942.67	0.36669	1.218 (1.166,1.272)	1.073 (1.027,1.121)	1.072 (1.026,1.12)	1.261 (1.194,1.333)
Blood pressure								
No	4,579,499	5,210	19,280,126.17	0.27023	1 (Ref.)	1 (Ref.)	1 (Ref.)	1 (Ref.)
Yes	3,747,454	5,817	15,829,140.23	0.36749	1.352 (1.302,1.403)	1.009 (0.969,1.051)	1.018 (0.977,1.06)	1.053 (1.01,1.097)
glucose								
No	5,508,624	6,739	23,248,480.56	0.28987	1 (Ref.)	1 (Ref.)	1 (Ref.)	1 (Ref.)
Yes	2,818,329	4,288	11,860,785.84	0.36153	1.243 (1.197,1.292)	1.034 (0.994,1.076)	1.038 (0.998,1.08)	1.059 (1.017,1.102)
HDL								
No	5,757,284	6,756	24,210,642.81	0.27905	1 (Ref.)	1 (Ref.)	1 (Ref.)	1 (Ref.)
Yes	2,569,669	4,271	10,898,623.59	0.39188	1.395 (1.343,1.45)	1.065 (1.023,1.109)	1.044 (1.003,1.087)	1.074 (1.031,1.119)
Triglyceride								
No	5,256,814	6,394	22,131,874.73	0.2889	1 (Ref.)	1 (Ref.)	1 (Ref.)	1 (Ref.)
Yes	3,070,139	4,633	12,977,391.67	0.35701	1.233 (1.187,1.28)	1.078 (1.038,1.121)	1.066 (1.025,1.108)	1.106 (1.062,1.151)

Model 1 was not adjusted. Model 2 was adjusted for age and sex. Model 3 was adjusted for age, sex, smoking, alcohol drinking, regular exercise, and income status. Model 4 was adjusted for age, sex, smoking, alcohol drinking, regular exercise, low-income status, baseline diabetes, hypertension, and dyslipidemia. Abbreviation: HDL, high-density lipoprotein. Metabolic syndrome was defined based on the National Cholesterol Education Program Adult Treatment Panel guideline (NCEP-ATP III) definition.

### 3.3 Risk of depression by variability of metabolic parameters

Those with highly variable metabolic parameters (i.e., 4th quartile) had a greater risk of having new onset BD compared to those with less variable metabolic parameters (i.e., 1st quartile) in all models (from model 1 to model 4) (Table 3). All metabolic parameters showed similar results. The number of highly variable metabolic parameters also showed a significant linear association with new onset BD (Figure 1 and Table 4). The association was significant in model 4 (for those with 5 highly variable metabolic parameters: HR = 2.5, 95% CI: 1.9–3.3).

**Table 3:** Hazard ratios and 95% confidence intervals of new onset bipolar disorder by quartiles of metabolic parameter variability.

	*N*	New onset bipolar disorder, *N*	Follow-up duration (person-year)	Incidence rate (per 1,000)	Hazard ratio (95% C.I)
Waist circumference variability					
Q1 (low variability)	2,081,959	2,376	8,775,871.3	0.2707	1 (Ref.)
Q2	2,081,536	2,442	8,804,684.7	0.2774	1.05 (0.99,1.11)
Q3	2,081,716	2,657	8,791,756.5	0.3022	1.12 (1.06, 1.19)
Q4 (high variability)	2,081,742	3,552	8,736,953.9	0.4066	1.45 (1.38, 1.53)
Glucose variability					
Q1	2,081,704	2,667	8,748,241.4	0.3049	1 (Ref.)
Q2	2,081,953	2,614	8,793,458.2	0.2973	1.02 (0.96,1.07)
Q3	2,081,567	2,728	8,797,715.1	0.3101	1.07 (1.01,1.13)
Q4	2,081,729	3,018	8,769,851.7	0.3441	1.14 (1.09,1.21)
Blood pressure variability					
Q1	2,080,439	2,516	8,732,855.5	0.2881	1 (Ref.)
Q2	2,082,934	2,403	8,788,390.4	0.2734	0.98 (0.92, 1.03)
Q3	2,081,356	2,599	8,810,867.6	0.2950	1.02 (0.97, 1.08)
Q4	2,082,224	3,509	8,777,153.0	0.3998	1.24 (1.17, 1.30)
HDL variability					
Q1	2,081,704	2,487	8,733,104.6	0.2848	1 (Ref.)
Q2	2,081,806	2,447	8,796,057.5	0.2782	0.98 (0.92,1.03)
Q3	2,081,708	2,662	8,805,692.1	0.3023	1.01 (0.96,1.07)
Q4	2,081,735	3,431	8,774,412.2	0.3910	1.17 (1.11,1.24)
Triglyceride variability					
Q1	2,081,738	2,805	8,738,630.0	0.3210	1 (Ref.)
Q2	2,081,738	2,611	8,796,146.2	0.2968	0.97 (0.92,1.03)
Q3	2,081,739	2,628	8,802,954.8	0.2985	0.99 (0.94,1.04)
Q4	2,081,738	2,983	8,771,535.5	0.3401	1.13 (1.07,1.19)

Variability was measured using variability independent of the mean (VIM) adjusted for age, sex, smoking, alcohol drinking, regular exercise, income status, baseline diabetes, hypertension, and dyslipidemia. HDL, high-density lipoprotein.

**Table 4:** Hazard ratios and 95% confidence intervals of new onset bipolar disorder by the number of highly variable (4th quartile) metabolic parameters.

Variables	*N*	Number of new onset bipolar disorder	Follow-up duration (Person-years)	Incidence rate (per 1,000 person-years)	Total			
					Model 1	Model 2	Model 3	Model 4
0	2,187,047	2,133	9239125.9	0.23087	1 (Ref.)	1 (Ref.)	1 (Ref.)	1 (Ref.)
1	3,084,330	3,826	13007881.9	0.29413	1.27 (1.21, 1.34)	1.22 (1.16, 1.29)	1.21 (1.15, 1.28)	1.21 (1.14, 1.27)
2	2,057,212	3,066	8666754.2	0.35377	1.53 (1.45, 1.62)	1.41 (1.33, 1.49)	1.39 (1.31, 1.47)	1.37 (1.30, 1.45)
3	800,920	1,528	3368293.4	0.45364	1.96 (1.83, 2.09)	1.74 (1.63, 1.86)	1.70 (1.59, 1.82)	1.67 (1.57, 1.79)
4	179,566	419	752419.7	0.55687	2.40 (2.16, 2.67)	2.06 (1.86, 2.29)	2.01 (1.81, 2.23)	1.96 (1.77, 2.18)
5	17,878	55	74791.4	0.73538	3.18 (2.43, 4.15)	2.64 (2.02, 3.45)	2.56 (1.96, 3.35)	2.49 (1.90,3.25)

Model 1 was not adjusted. Model 2 was adjusted for age and sex. Model 3 was adjusted for age, sex, smoking, alcohol drinking, regular exercise, and income status. Model 4 was adjusted for age, sex, smoking, alcohol drinking, regular exercise, low-income status, baseline diabetes, hypertension, and dyslipidemia.

### 3.4 Stratified analysis for pre-existing depression

**Figure 2:**
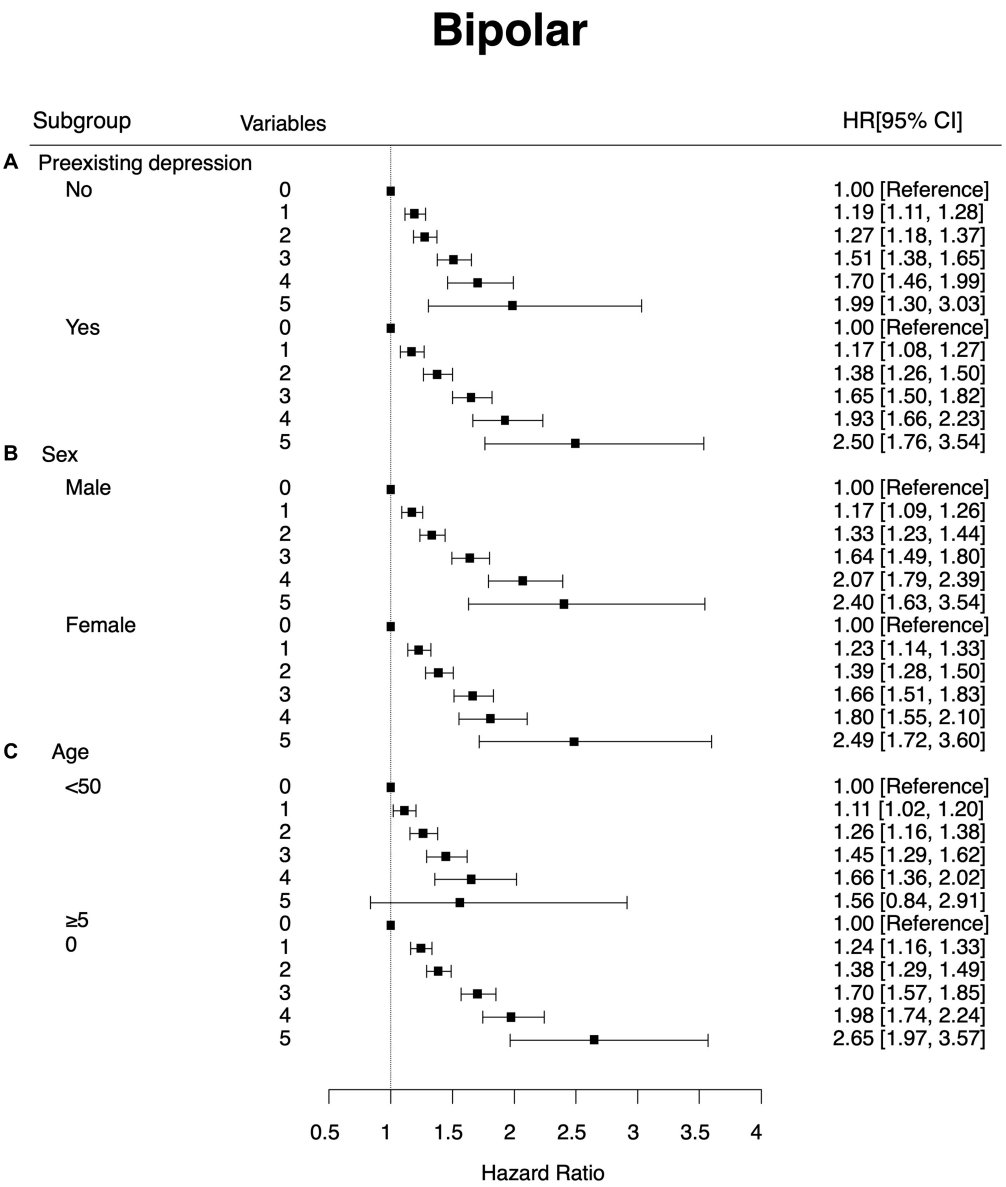
Subgroup analysis that showed hazard ratios and 95% confidence intervals of new onset bipolar disorder by the number of highly variable (4th quartile) metabolic parameters. The Y-axis refers to the number of highly variable metabolic parameters (highly variable metabolic parameters were defined as the 4th quartile of the variability of the parameter). Subgroup **(A)** by preexisting depression **(B)** by sex and **(C)** by age group.

As previously discussed, the experience of depression before the diagnosis of BD can be seen as a diagnostic switch from unipolar to BD. To adjust for the effect of pre-existing depression (F32 and F33), we conducted subgroup analyses depending on the history of pre-existing depression. In both groups, the risk of having diagnosis of BD increased as the number of highly variable metabolic parameters increased. No interaction effect was detected (Figure 2).

### 3.5 Effects of age and sex

Figure 1 shows the effect of sex on the association between newly developed BD and the number of highly variable metabolic parameters. Overall patterns and hazard ratios for metabolic parameters with high variability were similar between males and females (HR = 2.4, 95% CI: 1.6–3.5 for males with five highly variable metabolic parameters; HR = 2.5, 95% CI: 1.7–3.6 for females with five highly variable metabolic parameters).

We also divided participants into two groups based on their age with a cut-off of 50 years old. Overall patterns of hazard ratios for metabolic parameters with high variability were similar between those aged <50 years and those aged ≥50 years, although the hazard ratio was greater in those aged ≥50 years (HR: 1.67, 95% CI: 0.90–3.12 for those aged <50 years; HR: 2.65, 95% CI: 1.97–3.57 for those aged ≥50 years). No interaction effect was detected.

## 4 Discussion

This is the first study that demonstrates associations of metabolic syndrome and variability of metabolic parameters with the risk of developing BD. Using large-scale nationally representative data, we found that the presence of metabolic syndrome increased the risk of new onset BD. In addition, the variability in metabolic parameters increased the risk of new onset BD. Each metabolic parameter with a high variability showed a significant association with the risk of new onset BD. As the number of highly variable metabolic parameters increased, the risk for developing depression also increased even after adjusting for potential confounding factors.

In addition to metabolic syndrome, metabolic variability also showed significant associations with new onset BD, which corresponded with our previous study on metabolic variability and depression (9). Namely, metabolic variability could contribute to the development of mood spectrum disorder including both depression and BD.

The association between metabolic variability and newly developed BD was greater in old age group. Previous studies have suggested old age onset BD might have different neurological underpinning compared to typical BD (19). Namely, an old age onset BD was more frequently associated with neurological or physical conditions than a typical BD. Thus, metabolic variability could be more important in the development of BD during old age.

Under the DSM system, bipolar diagnosis in those with previous history of depression is not considered as a new onset BD but as a mood switch. In our study, the association between metabolic variability and new onset BD remained significant even after adjusting for the pre-existing depression. In the bipolar staging model, mood switch from unipolar depression to BD is considered as a stage progression that could be associated with neuro-progression (20).

Shared pathophysiology between metabolic syndrome and BD has been well established (21). Metabolic syndrome induces low-grade chronic inflammation (22). Metabolic overloads induce hypertrophic growth of adipose tissues. Adipocyte synthesizes and releases pro-inflammatory adipokine and cytokines. In addition, lipolysis of visceral fat and adipocyte rupture due to hypertrophy both could induce inflammation. Increased free fatty acid in circulating blood accumulates in other organs including liver, and fat accumulation in liver lead overproduction of low density (LDL), C-reactive protein (CRP) and interleukin (IL) -6. Fat also affect microbiata in intestine. Chronic inflammation is considered as a core pathophysiologic mechanisms of development of BD (23). Leptin, a peptide hormone secreted by adipocytes, has also been suggested as a possible link between obesity and mood disorder (24). Vascular endothelial dysfunction, increased oxidative stress, and increased sympathetic activation could also be associated with both obesity and BD (25). Finally, dopamine dysregulation might directly affect both obesity (4) and BD (26).

Like the association between metabolic variability and new onset depression, several hypotheses can be proposed on the relationship between the metabolic variability and new onset BD. Namely, metabolic variability could cause fluctuations in compositions of atherosclerotic plaques (27), eventually leading to plaque rupture (28). Microvascular lesions could impact brain regions involved in the pathogenesis of BD (8). Endothelial dysfunction related to metabolic variability could impact cerebral blood flow (29). Altered cerebral perfusion has also been observed in BD (30). In addition, fluctuations in metabolic parameters could affect the immune function (31). NK cell shows a significant association with white matter integrity in BD (32). Altered cholesterol metabolism can impact neuronal membrane function. Lastly, higher variabilities of multiple biological parameters have been observed in patients with systemic conditions and general frailty (33). Associations of frailty with severe mental illnesses have been reported (34).

However, the association observed in this study might be derived from the prodrome or early signs of untreated BD. Non-adherence to treatment for metabolic syndrome (35), significant appetite change (36), and sedentary lifestyle (37) could be due to untreated mood symptoms. Mood symptoms also are known to worsen metabolic syndrome (38) and the variability of metabolic parameters.

Our findings need to be interpreted considering limitations of the study design. First, we relied on ICD diagnostic code used for hospital visit when defining the development of BD. Second, we could not conclude the causal relationship based on our study findings. Third, the observation period for the development of BD might not be long enough to confirm the association.

In conclusion, this study demonstrated an association between metabolic variability and new onset BD. The metabolic syndrome and metabolic variability could be environmental modifiable risk factor for development of BD. Further prospective studies or intervention studies are needed to confirm our study findings.

## Data availability statement

The data analyzed in this study was obtained from the Korean National Health Insurance Service (NHIS). Datasets are available on request to the corresponding author.

## Ethics statement

The studies involving humans were approved by Institutional review board of Samsung medical center. The studies were conducted in accordance with the local legislation and institutional requirements. The ethics committee/institutional review board waived the requirement of written informed consent for participation from the participants or the participants’ legal guardians/next of kin because all data was de-identified before the statistical analyses.

## Author contributions

JHB: Writing – original draft. KH: Formal analysis, Writing – review & editing. HK: Writing – review & editing. KY: Writing – review & editing. HJ: Supervision, Writing – review & editing.
